# Emerging Molecular Links Between Plant Photomorphogenesis and Virus Resistance

**DOI:** 10.3389/fpls.2020.00920

**Published:** 2020-06-30

**Authors:** Ying Zhai, Hao Peng, Michael M. Neff, Hanu R. Pappu

**Affiliations:** ^1^Department of Plant Pathology, Washington State University, Pullman, WA, United States; ^2^Department of Crop and Soil Sciences, Washington State University, Pullman, WA, United States

**Keywords:** ATAF2, brassinosteroid, COP1, photomorphogenesis, photoreceptor, transcription factor, virus resistance

## Abstract

Photomorphogenesis refers to photoreceptor-mediated morphological changes in plant development that are triggered by light. Multiple photoreceptors and transcription factors (TFs) are involved in the molecular regulation of photomorphogenesis. Likewise, light can also modulate the outcome of plant–virus interactions since both photosynthesis and many viral infection events occur in the chloroplast. Despite the apparent association between photosynthesis and virus infection, little is known about whether there are also interplays between photomorphogenesis and plant virus resistance. Recent research suggests that plant–virus interactions are potentially regulated by several photoreceptors and photomorphogenesis regulators, including phytochromes A and B (PHYA and PHYB), cryptochromes 2 (CRY2), phototropin 2 (PHOT2), the photomorphogenesis repressor constitutive photomorphogenesis 1 (COP1), the NAM, ATAF, and CUC (NAC)-family TF ATAF2, the Aux/IAA protein phytochrome-associated protein 1 (PAP1), the homeodomain-leucine zipper (HD-Zip) TF HAT1, and the core circadian clock component circadian clock associated 1 (CCA1). Particularly, the plant growth promoting brassinosteroid (BR) hormones play critical roles in integrating the regulatory pathways of plant photomorphogenesis and viral defense. Here, we summarize the current understanding of molecular mechanisms linking plant photomorphogenesis and defense against viruses, which represents an emerging interdisciplinary research topic in both molecular plant biology and virology.

## Introduction

Light is a critical environmental factor for both plant growth (Wang et al., 2019) and virus infection (Paudel and Sanfaçon, 2018). On the plant side, light is the major environmental input for photosynthesis (Liu et al., 2019) as well as photomorphogenesis (Montgomery, 2016). Photomorphogenesis refers to a series of morphological changes in plant development when dark-grown seedlings are exposed to light (Paik and Huq, 2019). In the model plant *Arabidopsis thaliana*, seedling photomorphogenic changes include the cessation of hypocotyl elongation and the opening of cotyledons (von Arnim and Deng, 1996). There are also significant changes in gene expression underlying photomorphogenesis, which are regulated by multiple photoreceptors, phytochrome interacting factors (PIFs), and phytohormones (Wu, 2014). The light signal is overall an indicator of time and space for governing seedling development (Neff et al., 2000).

On the virus side, light intensity can modulate the outcome of plant–virus interactions since both photosynthesis and many viral infection events occur in the chloroplast (Li et al., 2016; Zhao et al., 2016; Bhattacharyya and Chakraborty, 2018). For example, high light intensity promotes the infection of clover by *Subterranean clover red leaf virus* (SCRLV) (Helms et al., 1987). *Arabidopsis* plants exhibit light-dependent hypersensitive response (HR) and resistance signaling against *Turnip crinkle virus* (TCV) (Chandra-Shekara et al., 2006). Both light deficiency and photosystem impairment can increase the susceptibility of *Nicotiana benthamiana* to *Turnip mosaic virus* (TuMV) infection (Manfre et al., 2011).

Despite the elucidation of multiple lines of evidence connecting photosynthesis and viral infection, the links between photomorphogenesis and plant–virus interactions have been elusive. Since photomorphogenesis also involves major changes in the ultrastructure of the plastids (Hills et al., 2015), the conversion from etioplasts to chloroplasts (Plöscher et al., 2011) would facilitate the replication of many viruses. Recently, there are reports that attribute several photoreceptors and photomorphogenesis regulators to the regulation of host–virus interactions, including phytochromes A and B (PHYA and PHYB); cryptochromes 2 (CRY2); phototropin 2 (PHOT2); the E3 ubiquitin ligase constitutive photomorphogenesis 1 (COP1) (Deng et al., 1992; Lim et al., 2018); the NAM, ATAF, and CUC (NAC)-family transcription factor (TF) ATAF2 (ANAC081) (Wang et al., 2009; Peng et al., 2015; Peng and Neff, 2020); the Aux/IAA protein phytochrome-associated protein 1 (PAP1) (Choi et al., 1999; Padmanabhan et al., 2005); the homeodomain-leucine zipper (HD-Zip) TF HAT1 (Sawa et al., 2002; Ciarbelli et al., 2008; Sorin et al., 2009; Zhang et al., 2014; Zou et al., 2016); and the MYB TF circadian clock associated 1 (CCA1) (Wang and Tobin, 1998; Zhai et al., 2019). Particularly, the plant growth promoting brassinosteroid (BR) hormones play critical roles in integrating photomorphogenesis and virus resistance. These findings suggest an overlap between plant photomorphogenesis and viral defense regulatory pathways.

## Photoreceptors PHYA, PHYB, CRY2, and PHOT2 Confer Virus Resistance

As two major phytochromes for photomorphogenesis induction, both PHYA and PHYB positively regulate plant defense against pathogens (Xie et al., 2011). In *Nicotiana tabacum*, PHYA and PHYB are essential for conferring resistance to *Cucumber mosaic virus* (CMV) (Li et al., 2015; Chen et al., 2018) and *Chilli veinal mottle virus* (ChiVMV) (Fei et al., 2019). The CMV genome consists of three single-stranded (ss) positive-sense (+) RNAs (Jacquemond, 2012). CMV replicates in the cytoplasm. Its three ss (+) RNAs have five open reading frames (ORFs) that encode a coat protein (CP), a movement protein (MP), and replication-relevant proteins such as the RNA-dependent RNA polymerase (RdRP), and serve as templates for the generation of complementary minus-sense (−) RNAs. These ss (−) RNAs are used to produce the ss (+) RNA CMV genome (Jacquemond, 2012). Belonging to the family *Potyviridae*, ChiVMV has an ss (+) RNA genome and also replicates in the cytoplasm following the (+) RNA virus replication model (Nigam et al., 2019).

In *Arabidopsis*, two blue light photoreceptors CRY2 and PHOT2 are indispensable for TCV resistance signaling mediated by hypersensitive response to TCV (HRT) (Jeong et al., 2010). The TCV ss (+) RNA genome has five ORFs, which encode two replication-related proteins p28 and p88, two MPs p8 and p9, and a CP (Carrington et al., 1989). TCV replication begins with the migration of p28 to the mitochondrial membrane and the binding of its ss (+) RNA genome to p28 (Hacker et al., 1992). Complementary ss (−) RNA and progeny ss (+) RNA production are similar to those of CMV and ChiVMV. HRT contains a coiled-coil nucleotide-binding site leucine-rich repeat (CC-NBS-LRR) motif (Cooley et al., 2000; Zhao et al., 2000), with its post-translational stability being maintained by double-stranded RNA binding proteins (DRBs) DRB1 and DRB4 (Zhu et al., 2013). The virus suppression activity of HRT is activated by TCV CP (Jeong et al., 2010). HRT is also named resistant to CMV(Y) 1 (RCY1) as it has another function in conferring CMV resistance (Takahashi et al., 2002; Sekine et al., 2008).

## The Photomorphogenesis Repressor COP1 Positively Regulates Plant Virus Resistance

Constitutive photomorphogenesis 1 was initially identified as a repressor of *Arabidopsis* photomorphogenesis in darkness, while its suppressing activity is reversed by light (Deng et al., 1992). Multiple light-activated photoreceptors, including PHYA, PHYB, CRY1, CRY2, PHOT2, and UVR8, interact with COP1 to suppress its function (Podolec and Ulm, 2018). As an E3 ubiquitin ligase, COP1 forms complexes with one of the four suppressor of phytochrome A-105 (SPA) proteins and ubiquitinates downstream TFs to mark them for degradation, which results in the suppression of photomorphogenesis (Hoecker, 2017). The degradation targets of COP1/SPA include two TFs HY5 (Osterlund et al., 2000) and LAF1 (Seo et al., 2003), both of which induce seedling de-etiolation and photomorphogenesis. In addition to photomorphogenesis, COP1 is also involved in the signaling pathways of major plant hormones, including auxin, ethylene, BR, gibberellin, jasmonic acid, abscisic acid, cytokinin, and strigolactone (Wang et al., 2019).

In contrast to its negative regulatory role in photomorphogenesis, COP1 positively regulates *Arabidopsis* resistance against TCV via modulating HRT activity and stability (Lim et al., 2018). Instead of directly interacting with CRY2 or PHOT2, HRT physically interacts with their interactor COP1 (Jeong et al., 2010). COP1 is an indispensable component in HRT-mediated resistance to TCV (Lim et al., 2018). COP1 also helps to stabilize DRB1 and DRB4, which are required for HRT stability (Lim et al., 2018). These results suggest that COP1 positively regulates TCV resistance in plants, which is consistent with an earlier observation that *Arabidopsis* exhibits light-dependent HR and resistance signaling against TCV (Chandra-Shekara et al., 2006).

## ATAF2 and PAP1 Integrate *Arabidopsis* Photomorphogenesis and Virus Resistance

ATAF2 was originally characterized as a transcriptional repressor of *Arabidopsis* pathogenesis-related genes (Delessert et al., 2005). *ATAF2* overexpression increases plant susceptibility to the fungal pathogen *Fusarium oxysporum* (Delessert et al., 2005). In addition to negatively regulating fungal resistance, ATAF2 is also involved in plant defense against *Tobacco mosaic virus* (TMV). The ss (+) RNA TMV was the first virus described (Scholthof, 2004). Its genome consists of four ORFs, which encode a replicase, an RdRP, a MP, and a CP (Lomonossoff and Wege, 2018). Similar to most (+) RNA viruses, TMV replicates in host cytoplasm via a (−) RNA intermediate (Scholthof, 2004). Transcriptionally induced by TMV infection, ATAF2 directly interacts with TMV replicase to induce resistance responses (Wang et al., 2009). Consistently, *ATAF2* overexpression can significantly reduce TMV accumulation in plants (Wang et al., 2009). As a counteraction, TMV replicase protein can physically interact with ATAF2 via its helicase domain and consequently promote virus accumulation (Wang et al., 2009). The interaction may also facilitate targeted degradation of ATAF2 during TMV infection (Wang et al., 2009).

ATAF2 is a repressor of *Arabidopsis* seedling photomorphogenesis (Peng et al., 2015). When grown in low-intensity white light, *ATAF2* loss- and gain-of-function seedlings exhibit hypocotyl lengths that are shorter and longer than those of the wild type, respectively (Peng et al., 2015). The far-red photoreceptor PHYA plays a major role in ATAF2-regulated photomorphogenesis and is required for the suppression of *ATAF2* expression in continuous light (Peng et al., 2015). PHYA also physically interacts with an Aux/IAA protein PAP1 (Choi et al., 1999), but the impact of this interaction on photomorphogenesis is still unclear. Also known as IAA26, PAP1 is thought to heterodimerize with auxin response factors (ARFs) and act as a repressor of auxin-induced gene expression (Padmanabhan et al., 2005). Like ATAF2, PAP1 also physically interacts with TMV replicase (Padmanabhan et al., 2005). The interaction disrupts PAP1 localization, disturbs the *Arabidopsis* auxin response system, and thereby facilitates TMV infection (Padmanabhan et al., 2005).

## Integration of Photomorphogenesis and Virus Resistance Via the Plant Growth-Promoting Brassinosteroid Hormones

ATAF2-regulated hypocotyl growth phenotypes are closely related to BR homeostasis (Peng et al., 2015). BRs modulate *Arabidopsis* seedling photomorphogenesis via promoting hypocotyl growth under light but inhibiting its elongation in the dark (Turk et al., 2003). ATAF2 promotes BR accumulation via suppressing the expression of two BR-inactivating cytochrome P450 genes *BAS1* (*CYP734A1*, formerly *CYP72B1*) and *SOB7* (*CYP72C1*) (Neff et al., 1999; Turk et al., 2005), and therefore plays a critical role in BR-regulated photomorphogenesis (Peng et al., 2015).

Brassinosteroids themselves actually function in a broad range of plant disease resistance (Nakashita et al., 2003), including defense responses against multiple viral pathogens such as TMV in *N. benthamiana* (Deng et al., 2016), CMV in *Arabidopsis* (Zhang et al., 2015; Zou et al., 2018), *Sweet potato leaf curl virus* (SPLCV) in *Arabidopsis* (Bi et al., 2017), *Rice black-streaked dwarf virus* (RBSDV) in rice (He et al., 2017; Zhang et al., 2019), *Maize chlorotic mottle virus* (MCMV) in maize (Cao et al., 2019), and *Tomato yellow leaf curl virus* (TYLCV) in *Arabidopsis* and *N. benthamiana* (Garnelo Gómez et al., 2019). BRs may play either positive or negative roles in plant viral defense depending on virus species. As a modulator of BR homeostasis, ATAF2 may be indirectly involved in BR-regulated virus defense in *Arabidopsis*.

The photomorphogenesis and TCV repressor COP1 is reported to physically interact and suppress core BR signaling proteins, including brassinosteroid-insensitive 2 (BIN2) (Ling et al., 2017), phosphorylated brassinazole-resistant 1 (pBZR1) (Kim et al., 2014), and GATA transcription factor 2 (GATA2) (Luo et al., 2010).

*Arabidopsis* HD-Zip TF HAT1 is another molecular link connecting BRs, the phytochrome system, and plant virus resistance. HAT1 is directly regulated by phytochromes (Sawa et al., 2002) and plays a positive role in shade avoidance (Sorin et al., 2009). Consistently, *HAT1* transcript accumulation is induced by low red/far-red (R/FR) ratio light and suppressed in high R/FR (Ciarbelli et al., 2008). HAT1 interacts with BRI1-EMS-suppressor 1 (BES1) to cooperatively inhibit BR-repressed gene expression via direct promoter binding (Zhang et al., 2014). In another report, HAT1 negatively regulates *Arabidopsis* response to CMV with its gain- and loss-of-function mutants exhibiting susceptible and resistant phenotypes, respectively (Zou et al., 2016).

ATAF2 also interacts with the core circadian regulator CCA1 both physically and genetically to suppress *BAS1* and *SOB7* expression (Peng and Neff, 2020). CCA1 is a MYB-family TF that regulates *Arabidopsis* circadian rhythms (Wang and Tobin, 1998) together with its partially redundant paralog late elongated hypocotyl (LHY) (Schaffer et al., 1998; Mizoguchi et al., 2002). CCA1 binds to the promoters of its target genes via recognizing the evening element (EE) or the CCA1-binding site (CBS) motifs (Michael and McClung, 2002). CCA1 can bind to the CBS-containing promoter of *ATAF2* and suppress its transcript accumulation in the light, whereas CCA1 acts as an activator of *ATAF2* transcription in the dark (Peng and Neff, 2020). CCA1 and ATAF2 physically interact with each other and both bind the EE/CBS motifs of *BAS1* and *SOB7* promoters to suppress their expression (Peng and Neff, 2020). In addition to these two BR catabolic genes, CCA1 can activate the expression of the key BR biosynthetic gene *DWF4* (*CYP90B1*) via direct binding to its promoter (Zheng et al., 2018). Taken together, CCA1 is a positive regulator of BR accumulation, indicating its similar role as ATAF2 in BR-regulated virus defense.

## The Core Circadian Clock Component Cca1 Suppresses Photomorphogenesis and May Bind Virus-Derived Sequence Directly

As a core regulatory component of circadian oscillation, CCA1 is also involved in the photomorphogenic pathways, which was initially proposed from the genetic observation that the *cca1 lhy* double mutant displays hypersensitivity to red light during early photomorphogenesis (Ito et al., 2007). Subsequent biochemical evidence demonstrated the physical interactions between CCA1 and core photomorphogenic proteins such as de-etiolated 1 (DET1) (Chory et al., 1989) and COP1 suppressor 4 (CSU4) (Zhao et al., 2018). The COP10-DET1-DDB1 (CDD) complex is an evolutionarily conserved protein complex that suppresses photomorphogenesis in *Arabidopsis* (Lau et al., 2011). Additionally, DET1 can physically interact with CCA1 and LHY to co-suppress their target genes (Lau et al., 2011). The recently identified CSU4 is a genetic suppressor of photomorphogenic repressors COP1 and DET1 (Zhao et al., 2018). CSU4 can suppress the transcriptional repression activity of CCA1 via physical interaction and negatively regulates the transcriptional expression of *CCA1* in the early morning (Zhao et al., 2018).

In additional to its indirect role in virus defense via interacting with ATAF2/COP1 and modulating BR homeostasis, CCA1 may bind virus-derived sequences directly (Zhai et al., 2019). CCA1 binds more than 1000 EE/CBS motifs in the *Arabidopsis* genome (Nagel et al., 2015; Kamioka et al., 2016). It is possible that CCA1 may bind not only the EE/CBS-containing promoters of plant genes but also similar sequences from invading pathogens. Recent evidence suggests that CCA1 may interact with the DNA form of *Tomato spotted wilt tospovirus* (TSWV) genome (Zhai et al., 2019). TSWV has three ssRNAs designated as large (L), medium (M), and small (S) RNAs. The L RNA is (−) while both M and S RNAs are ambisense. The 5′-upstream region of the TSWV G_N_/G_C_ gene in M RNA harbors a CBS motif that can directly bind CCA1 when expressed as cDNA (Zhai et al., 2019). Disruption of this CBS motif is sufficient to switch the promoter activity from light suppressive to light inducible (Zhai et al., 2019). Since the whole life cycle of TSWV is completed in the cytoplasm (Zhai et al., 2014), whether CCA1 can bind the native RNA form of G_N_/G_C_ is questionable. The observation that CCA1 can be detected in both cytoplasm and nucleus (Yakir et al., 2009) makes CCA1-TSWV interaction spatially possible. However, there is still no evidence that CCA1 influences TSWV infectivity. The first report of an infectious TSWV clone (Feng et al., 2020) may help to elucidate whether CCA1 can interact with the native TSWV M RNA *in planta*.

## Summary of Current Understanding and Future Perspectives

**FIGURE 1:**
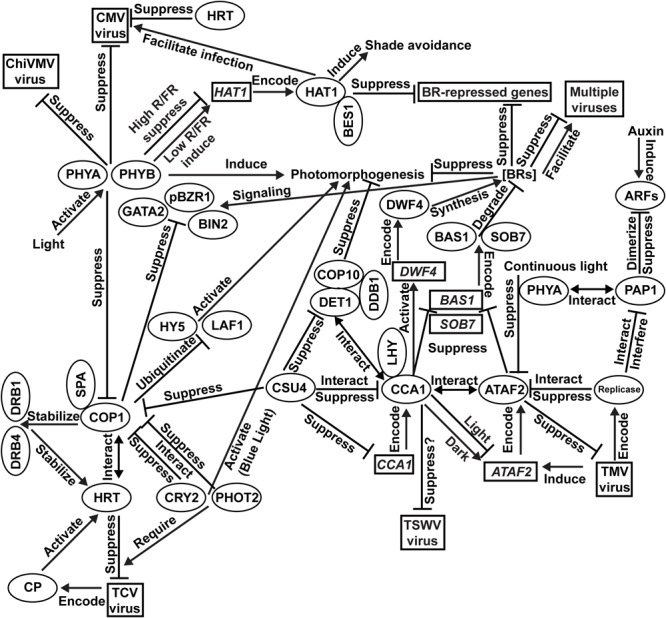
Integration of photomorphogenesis and virus resistance. Results of PHYA/PHYB-mediated resistance to *Cucumber mosaic virus* (CMV) and *Chilli veinal mottle virus* (ChiVMV) come from *Nicotiana tabacum*. All other findings are based on investigations using *Arabidopsis*.

Our current understanding of the integration of photomorphogenesis and virus resistance is shown in Figure 1. Results of PHYA/PHYB-mediated resistance to CMV and ChiVMV come from *N. tabacum*. All other findings are based on investigations using *Arabidopsis*. Light activates multiple photoreceptors, including two phytochromes PHYA and PHYB, to induce plant photomorphogenesis. Both PHYA and PHYB are essential for plant defense against CMV and ChiVMV. The blue-light photoreceptors CRY2 and PHOT2 promote photomorphogenesis via interacting with COP1 to suppress its function. TCV-encoded CP protein activates the HRT-mediated TCV-resistance signaling pathway with the required participation of CRY2 and PHOT2. HRT also confers CMV resistance. The COP1/SPA complex suppresses photomorphogenesis via the ubiquitination and consequent degradation of photomorphogenic activators such as HY5 and LAF1. In the HRT-mediated virus-resistance signaling pathway, COP1 stabilizes HRT via direct interaction as well as stabilizing DRB1 and DRB4, which are required for the stabilization of HRT. Transcriptionally induced by TMV infection, ATAF2 suppresses TMV accumulation. TMV-encoded replicase can interact with ATAF2 and suppress its anti-TMV function. The presence of PHYA is required for ATAF2-mediated suppression of photomorphogenesis. PHYA physically interacts with auxin response repressor PAP1. TMV replicase physically interacts with PAP1 to interfere with its function. ATAF2 negatively regulates photomorphogenesis via suppressing two BR-catabolic genes *BAS1* and *SOB7*. BRs suppress photomorphogenesis under light and positively/negatively regulate viral resistance depending on virus species. COP1 physically interacts with and suppresses core BR signaling proteins BIN2, pBZR1, and GATA2. Directly regulated by phytochromes in an R/FR-ratio dependent manner, HAT1 induces shade avoidance and interacts with BES1 to cooperatively inhibit BR-repressed gene expression. HAT1 facilitates CMV infection. CCA1 physically interacts with ATAF2 protein and also regulate its transcription. CCA1 promotes BR accumulation by suppressing *BAS1*/*SOB7* and activating the expression BR-biosynthetic gene *DWF4*. CCA1 suppresses photomorphogenesis via the CSU4/COP1/COP10/DET1/DDB1 signaling network. CSU4 promotes photomorphogenesis by genetically suppressing COP1 and DET1. DET1 forms a photomorphogenesis-repressor complex with COP10 and DDB1. DET1 can also interact with CCA1/LHY to co-suppress downstream genes. CCA1 is suppressed by CSU4 at both transcription and protein–protein interaction levels. It is possible that CCA1 can bind the un-translated region of the TSWV genome and suppress virus accumulation, but direct evidence still lacks.

Photomorphogenesis is modulated by multiple photoreceptors and TFs, which regulate a large number of downstream genes to facilitate the plant’s transition from dark-grown to light-grown status. Meanwhile, plant susceptibility to virus infection may be affected by its developmental and physiological changes during photomorphogenesis. Therefore, it is not surprising that multiple photoreceptors and/or their regulatory targets are involved in virus resistance. For example, PHYA, PHYB, CRY2, and PHOT2 are all essential for plant defense against certain viruses. Two photomorphogenesis repressors, COP1 and ATAF2, are both virus suppressors and involved in BR signaling/metabolic pathways. Due to the diversity of plant virus species and the complexity of photomorphogenesis regulatory network and virus resistance signaling pathways, our knowledge on the links between plant photomorphogenesis and interactions with viruses is still limited. High-throughput screening of photomorphogenesis-related genes and proteins that are responsive to virus infection may reveal additional insights in the future.

## Author Contributions

All authors listed have made a substantial, direct and intellectual contribution to the work, and approved it for publication.

## Conflict of Interest

The authors declare that the research was conducted in the absence of any commercial or financial relationships that could be construed as a potential conflict of interest.
